# A Rare Case of Cutaneous Extramedullary Hematopoiesis in Chronic Myeloid Leukemia

**DOI:** 10.1111/cup.70114

**Published:** 2026-04-10

**Authors:** Bennett Christie‐Nguyen, Hanadi El Achi, Levani Iashvili, Amer Wahed, Brenda Mai, Jacob Armstrong

**Affiliations:** ^1^ Department of Pathology and Laboratory Medicine The University of Texas Health Science Center at Houston, McGovern Medical School Houston Texas USA

## Abstract

Cutaneous extramedullary hematopoiesis (CEH) is a rare manifestation of extramedullary hematopoiesis (EMH), a process typically associated with fetal development or myeloproliferative neoplasms. EMH most commonly involves the spleen, liver, and lymph nodes, with CEH being exceedingly rare in chronic myeloid leukemia (CML). Here, we present a 34‐year‐old Hispanic male with multiple bilateral lower extremity lesions, right buttock and leg pain, fatigue, and splenomegaly, who was found to have chronic‐phase CML. Punch biopsy of one of the skin lesions demonstrated dermal infiltration by maturing hematopoietic elements, including granulocytes, megakaryocytes, and erythroid precursors, with fluorescence in situ hybridization (FISH) testing confirming a neoplastic extramedullary hematopoietic infiltrate with the presence of *BCR::ABL1* fusion. Initial treatment included hydroxyurea and dasatinib; however, after chronic‐phase CML was confirmed, therapy was transitioned to imatinib, resulting in resolution of B symptoms, normalization of WBC counts, and reduced leg swelling. This case underscores the importance of distinguishing CEH from aggressive disease states, such as blast‐phase CML or myeloid sarcoma, through comprehensive histopathological and immunohistochemical analysis.

## 1 Introduction

Extramedullary hematopoiesis (EMH) refers to the production of blood cells outside the bone marrow, often seen in fetal development or myeloproliferative disorders. This process is typically reactive in nature, developing as a compensatory response to stress or bone marrow compromise often due to fibrosis or other space‐occupying lesions in the marrow.

While EMH most often occurs in the spleen, liver, and lymph nodes, involvement of the skin is less frequent [1]. Cutaneous extramedullary hematopoiesis (CEH) is well‐documented in primary myelofibrosis but rare in CML, with only a few reported cases. The presence of cutaneous lesions in the setting of myeloid neoplasms also raises other considerations, such as myeloid sarcoma (MS) and sclerosing extramedullary hematopoietic tumor (SEMHT) [2]. Here, we present a case of CEH representing cutaneous involvement by CML, thus termed “neoplastic CEH”, to describe the presence of neoplastic, but maturing, trilineage hematopoietic elements and to further distinguish from MS.

## 2 Case Reports

A 34‐year‐old Hispanic man presented with a two‐week history of right buttock and leg pain, multiple bilateral lower extremity “pustules”, fatigue, chills and night sweats. He had reportedly been evaluated 8 months earlier at another hospital, where a bone marrow biopsy was performed leading to a diagnosis of leukemia; however, he was unable to follow up with an oncologist due to financial constraints. A CT scan of the pelvis with contrast revealed splenomegaly and notable enlargement of the right gluteus maximus and medius muscles, with heterogeneous fluid collections suggestive of intramuscular hemorrhage, hematoma, infectious process, or myositis. Notably, a similar lesion to those involving the bilateral lower extremities was initially present at this site, reportedly treated with antibiotic injection prior to presentation to our facility. Upon admission, the patient was noted to have multiple erythematous, indurated plaques with central eschars involving the bilateral lower extremities, an elevated white blood count (WBC) of 388.2 k/μL and hemoglobin of 5.1 g/dL. Peripheral blood examination showed marked neutrophilic leukocytosis, basophilia and eosinophilia. A subsequent bone marrow biopsy demonstrated hypercellularity (> 90%), characterized by left‐shifted granulocytic hyperplasia, megakaryocytic hyperplasia, erythroid hypoplasia, and < 5% blasts, consistent with chronic‐phase CML. Chromosome analysis demonstrated a male karyotype with t(9;22) (q34.1;q11.2), and RT‐PCR confirmed the presence of the *BCR::ABL1* fusion gene with p210 fusion transcripts.

**FIGURE 1:**
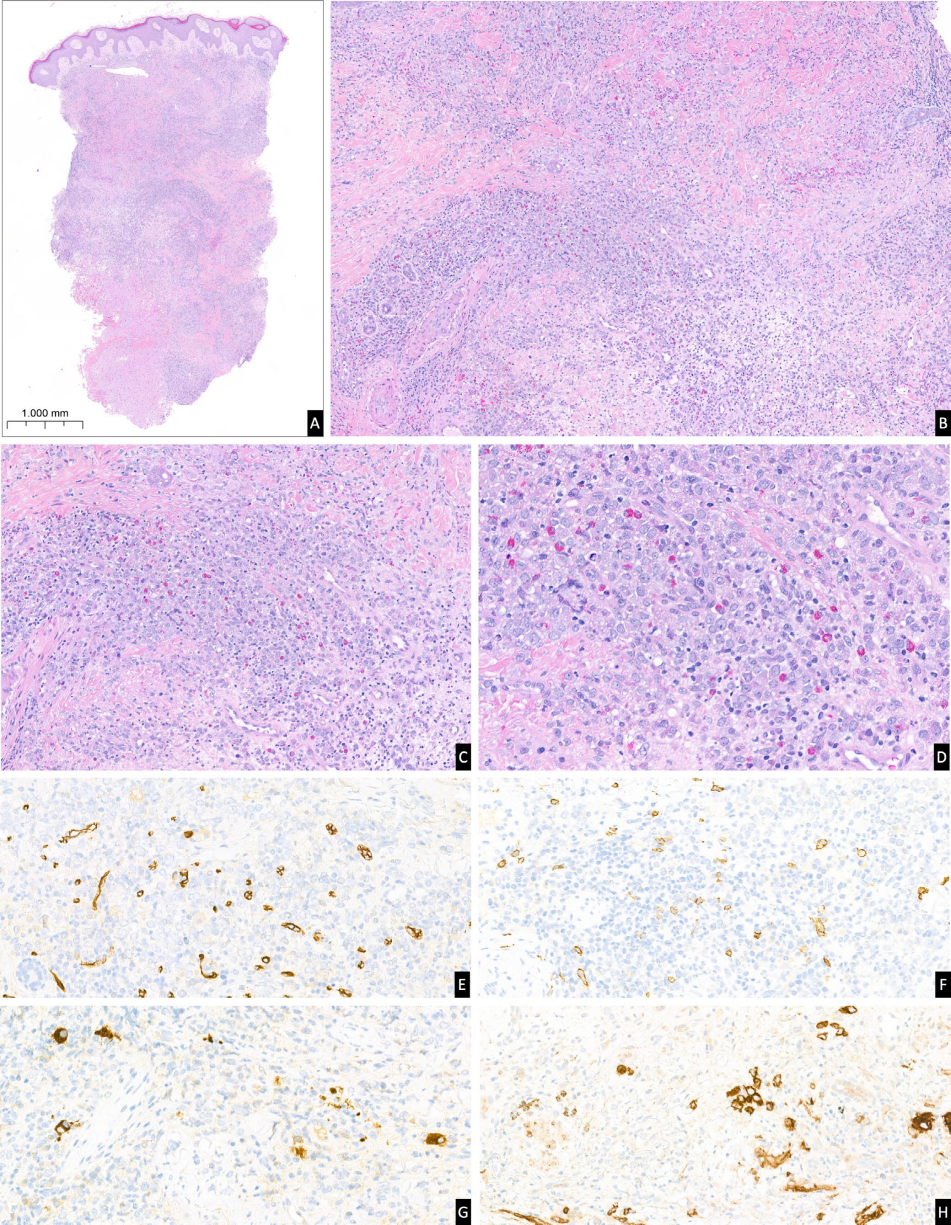
Cutaneous extramedullary hematopoiesis in chronic‐phase chronic myeloid leukemia. (A) Diffuse dermal infiltrate with an epidermal Grenz zone (H&E, 10×). (B–D) Higher‐power views show the mixed infiltrate of maturing granulocytes, megakaryocytes, and erythroid precursors, highlighting the abundance of left‐shifted cells (H&E; B: 100×, C: 200×, D: 400×). Immunohistochemistry: (E) CD34 shows no increase in blasts (100×, intensely stained areas are vascular structures), (F) CD117 highlights mast cells and rare immature cells (100×), (G) CD61 highlights megakaryocytes (100×), and (H) CD71 highlights erythroid precursors (100×).

A punch biopsy of a right lateral thigh lesion revealed a dermal infiltrate with epidermal sparing (Grenz zone). The lesional infiltrate was comprised of primarily left‐shifted but maturing granulocytes, along with scattered megakaryocytes and rare admixed erythroid precursors (Figure 1A–D). CD34 immunohistochemical staining (Figure 1E) showed no increase in blasts, while CD117 immunohistochemical staining predominantly highlighted scattered mast cells and only rare immature cells (Figure 1F). Immunohistochemical staining for CD61 (Figure 1G) and CD71 (Figure 1H) highlighted a few admixed megakaryocytes and erythroid precursors, respectively. Given the presence of trilineage hematopoietic elements, the overall features were consistent with maturing EMH, without an increase in or clusters of blasts suggestive of a MS and without fibrosis or significant megakaryocytic atypia suggestive of SEMHT. FISH for *BCR::ABL1* was performed on the skin biopsy specimen, revealing approximately 70% of the examined nuclei carried the *BCR::ABL1* fusion and confirming neoplastic origin of the EMH infiltrate.

The patient initially received hydroxyurea for cytoreduction, followed by dasatinib, a second‐generation tyrosine kinase inhibitor (TKI) based on the initial clinical impression of CML in a progressive stage due to the cutaneous manifestation. After confirmation of chronic‐phase CML, the patient was transitioned to imatinib, a first‐generation TKI. The response to treatment was favorable, with resolution of B symptoms, normalization of the WBC count, and improvement of the cutaneous lesions.

## 3 Discussion

CEH is a natural process occurring from the first through the fifth month of gestation. During this time, undifferentiated mesenchymal cells in the dermis retain their ability to produce hematopoietic cells. After the sixth month of gestation, hematopoiesis in the dermis typically ceases, except in certain conditions such as myeloproliferative neoplasms (MPN) [1].

While CEH is well‐documented in primary myelofibrosis (PMF), it is frequently described as exceedingly rare in CML [3]. In our initial PubMed search using the keywords “extramedullary hematopoiesis” and “chronic myeloid leukemia” or “chronic myelogenous leukemia”, we identified only four cases of CML presenting with EMH in the skin, while the others involved various other sites [4, 5, 6]. However, an expanded search using broader terms such as “skin” or “cutaneous” combined with “chronic myeloid leukemia” or “chronic myelogenous leukemia” reveals that specific skin involvement occurs in approximately 2% to 5% of patients [7, 8].

Many of these cases, though often labeled as leukemia cutis or even myeloid sarcoma, share the histological characteristics of maturing EMH. For example, Kaddu et al. evaluated nine patients with CML and found that seven biopsy specimens exhibited a pleomorphous infiltrate composed of a variable mixture of mature and immature cells of the granulocytic series [7] [Correction added on 17 April 2026, after first online publication: The preceding in‐text citation has been corrected in this version.]. Similarly, Afrose et al. described a patient with chronic‐phase CML whose penile foreskin showed an infiltrate of myeloid cells at different stages of maturation with few immature forms [9]. Vasconcelos et al. also reported a CML case where the skin biopsy revealed a dense mixed infiltrate of neutrophils, lymphocytes, and atypical cells specifically accompanied by megakaryocytes [10]. These findings suggest that a significant subset of CML involving the skin is characterized by a neoplastic, but maturing hematopoietic process rather than a predominantly or purely blastic one. It is critical to distinguish these maturing processes from true extramedullary blast crisis, which signifies an aggressive disease course. For instance, Madhumathi et al. reported a case of CML involving multiple cutaneous masses ultimately diagnosed as a lymphoid blast crisis. In that case, despite the presence of some myeloid cells, groups of blasts were noted, and the patient had 70% blasts in the peripheral smear and a rapid clinical decline [11].

Despite the variable presentations of CML in the skin, ours is the first reported case to confirm the *BCR::ABL1* fusion directly within the lesional cells of maturing CEH by FISH. This confirmation is significant because previous reports of subcutaneous masses with maturation in CML either failed to detect the fusion at the lesion site or lacked molecular confirmation of a neoplastic origin [12]. The terminology for these findings remains inconsistent across institutions, and there is no clearly agreed‐upon diagnosis for maturing neoplastic myeloid infiltrates. While we use the term “cutaneous extramedullary hematopoiesis” to highlight the trilineage, maturing nature of the cells, others may diagnose these same features as “cutaneous involvement by chronic myeloid leukemia” or simply “leukemia cutis”, though the latter is particularly ambiguous. Regardless of the exact terminology used, it is important to distinguish this from myeloid sarcoma, a term reserved for mass‐forming lesions with increased blasts or, in the context of monocytic differentiation, promonocytes, that often herald an aggressive disease course.

CEH can manifest as either a reactive process or a sign of disease progression, with neoplastic cells identified within the skin lesions [13]. In clinical practice, CEH may be mistaken for more aggressive conditions, particularly in patients with myeloid neoplasms such as CML. CML has classically been recognized as having three phases of disease: chronic phase, accelerated phase and blast phase, though current classification systems are conflicted with regards to recognition of a separate “accelerated” phase in the context of current therapeutic regimens. Most patients present in chronic phase, and a subset progress to or present in blast phase, which is defined by the presence of ≥ 20% blasts in the peripheral blood or bone marrow or by extramedullary proliferation of blasts that disrupts tissue architecture, akin to MS [14]. CML presenting with MS often signifies clonal evolution, indicating an aggressive disease course and poor prognosis, and is thus considered blast phase regardless of bone marrow or peripheral blood blast counts [15, 16]. Consequently, the presence of skin lesions in CML patients often raises critical concerns, including MS/blast‐phase CML or Sweet syndrome. Each of these conditions has distinct clinical implications and requires individualized therapeutic approaches.

In MPNs, insufficient bone marrow capacity to produce adequate hematopoietic elements may arise from factors such as myelofibrosis, excessive proliferation of marrow components, or disruption of the bone marrow microenvironment. These conditions often lead to increased circulation of hematopoietic stem cells, which can migrate to extramedullary sites [17]. Additionally, aberrant cytokines or growth factors can drive stem cells to differentiate into hematopoietic lineages in ectopic locations, effectively creating a microenvironment mimicking that of bone marrow [18]. Although EMH often represents a compensatory process, its presentation in the context of MPNs may be neoplastic rather than reactive. One study described a case of CEH related to PMF, characterized as “cutaneous myelofibrosis”, where the presence of a *JAK2* mutation confirmed the lesion as a neoplastic myeloid proliferation consistent with SEMHT [19]. Cutaneous involvement in myeloid neoplasms exhibits a variable spectrum, including sheets of blasts effacing the normal histologic architecture as seen in MS, trilineage hematopoietic elements with megakaryocytic atypia and fibrosis as seen in SEMHT, and EMH. EMH is characterized by the proliferation of normal‐appearing hematopoietic elements with often multilineage proliferation, with erythroid, myeloid and/or megakaryocytic elements that lack dysplastic changes. Immature cells are rare, with a predominance of maturing or mature hematopoietic elements. Immunohistochemically, EMH shows minimal or no expression of markers of immaturity, such as CD34, CD117 or TdT, distinguishing it from lymphoblastic or MS, and lacks the fibrosis and significant megakaryocytic atypia described in SEMHT. Regardless of normal trilineage maturation, CEH/EMH associated with MPNs is likely a neoplastic process, mirroring its counterpart in the bone marrow. In contrast, EMH related to non‐hematolymphoid neoplasms is more likely a benign process [20].

In our case, skin biopsy demonstrated CEH with granulocyte‐predominant maturing trilineage hematopoietic elements lacking a significant increase in blasts or significant cytologic atypia, inconsistent with MS/blast‐phase of CML. Most significantly, the detection of *BCR::ABL1* fusion in the dermal lesion by FISH supports a neoplastic process. The patient responded well to imatinib, with resolution of B symptoms, normalization of WBC count, and improvement of the skin lesions, further confirming that the CEH in our case represented a neoplastic proliferation.

In summary, CEH in CML may be neoplastic or non‐neoplastic and, while rare, should be carefully distinguished from aggressive disease such as MS signifying blast phase of CML. Accurate diagnosis, supported by histopathology and IHC, allows for appropriate management focused on TKI therapy for CML, without escalating to intensive chemotherapy.

## Ethics Statement

The authors have nothing to report.

## Conflicts of Interest

The authors declare no conflicts of interest.

## Data Availability

The data that support the findings of this study are available on request from the corresponding author. The data are not publicly available due to privacy or ethical restrictions.
